# Single-cell analysis of human prepuce reveals dynamic changes in gene regulation and cellular communications

**DOI:** 10.1186/s12864-023-09615-8

**Published:** 2023-09-01

**Authors:** Fei Tan, Yuan Xuan, Lan Long, Yang Yu, Chunhua Zhang, Pengchen Liang, Yaoqun Wang, Meiyu Chen, Jiling Wen, Geng Chen

**Affiliations:** 1 School of Medicine, Shanghai Skin Disease Hospital, Tongji University, Shanghai, 200443 China; 2 Shanghai Skin Disease Clinical College, The Fifth Clinical Medical College, Anhui Medical University, Shanghai Skin Disease Hospital, Shanghai, 200443 China; 3 Longgang District Maternity & Child Healthcare Hospital of Shenzhen City, Shenzhen, 518172 China; 4 Department of Urology, Shanghai Tenth People’s Hospital, Tongji University School of Medicine, Shanghai, 200072 China; 5 Department of Dermatology, Shanghai Baoshan Hospital of Integrated Traditional Chinese and Western Medicine, Shanghai, 201999 China; 6 School of Microelectronics, Shanghai University, Shanghai, 201800 China; 7 Department of Urology, Shanghai East Hospital, Tongji University School of Medicine, Shanghai, 200120 China; 8 Center for Bioinformatics and Computational Biology, School of Life Sciences, East China Normal University, Shanghai, 200241 China

**Keywords:** Single-cell RNA-seq, Human prepuce, Gene regulation, Cell–cell communications, Cellular dynamics

## Abstract

**Background:**

The cellular and molecular dynamics of human prepuce are crucial for understanding its biological and physiological functions, as well as the prevention of related genital diseases. However, the cellular compositions and heterogeneity of human prepuce at single-cell resolution are still largely unknown. Here we systematically dissected the prepuce of children and adults based on the single-cell RNA-seq data of 90,770 qualified cells.

**Results:**

We identified 15 prepuce cell subtypes, including fibroblast, smooth muscle cells, T/natural killer cells, macrophages, vascular endothelial cells, and dendritic cells. The proportions of these cell types varied among different individuals as well as between children and adults. Moreover, we detected cell-type-specific gene regulatory networks (GRNs), which could contribute to the unique functions of related cell types. The GRNs were also highly dynamic between the prepuce cells of children and adults. Our cell–cell communication network analysis among different cell types revealed a set of child-specific (e.g., CD96, EPO, IFN-1, and WNT signaling pathways) and adult-specific (e.g., BMP10, NEGR, ncWNT, and NPR1 signaling pathways) signaling pathways. The variations of GRNs and cellular communications could be closely associated with prepuce development in children and prepuce maintenance in adults.

**Conclusions:**

Collectively, we systematically analyzed the cellular variations and molecular changes of the human prepuce at single-cell resolution. Our results gained insights into the heterogeneity of prepuce cells and shed light on the underlying molecular mechanisms of prepuce development and maintenance.

**Supplementary Information:**

The online version contains supplementary material available at 10.1186/s12864-023-09615-8.

## Introduction

The prepuce, commonly referred to as the foreskin, comprises a specialized tissue structure that encapsulates the glans penis in males [1–3]. Despite its evident biological and physiological significance, our understanding of the prepuce's cellular heterogeneity and functional diversity remains limited. With the advent of single-cell RNA-sequencing (scRNA-seq) technologies, gene/transcript expression profile is feasible to be investigated at a single-cell resolution [4, 5]. Moreover, scRNA-seq also enables the investigation of cellular expression heterogeneity, inference of gene regulatory networks, and construction of cell-to-cell interactions [6–9]. Although several studies have investigated the transcriptional profile of human epidermis (e.g., truncal skin, and scalp) at the single-cell level [10–12], the cellular composition and heterogeneity of human prepuce were still largely unknown. Single-cell analysis of human prepuce is essential for unraveling its complex tissue composition and physiological functions.

Furthermore, with the development of prepuce, its cell composition and gene expression profile could be dynamically changed. The expression heterogeneity of prepuce cells is largely correlated with the composition of cell subtypes, while expression profiles of cell subpopulations are directly influenced by the gene regulatory networks (GRNs) formed by transcription factors (TFs) and downstream target genes [13–15]. Additionally, different cell types usually interact with each other to exert corresponding functions, which are mediated by related ligands and receptors [16–20]. Thus, cellular differences in human prepuce between children and adults could be closely correlated to the variations of GRNs and cell–cell interaction networks. Systematic investigation of the prepuce's cellular dynamics and molecular changes could provide a deeper understanding of the role of different cell types in maintaining prepuce health and preventing infections. It could also add valuable knowledge to the fields of human anatomy, biology, and reproductive health.

Here, we first explored the expression heterogeneity and cell type compositions of human prepuce of children and adults based on related scRNA-seq data. Then, we constructed the gene regulatory networks (GRNs) for the prepuce cells and investigated the gene regulatory dynamics among transcription factors and downstream target genes. The GRN differences between children and adults were also examined. Additionally, we built the cellular interaction networks among different cell subtypes and interrogated cell–cell communication variations between children and adults. The signaling pathways enriched by the ligand-receptor pairs of cell–cell interactions were explored as well.

## Results

### Cell type identification for human prepuce based on scRNA-seq data

**Fig. 1:**
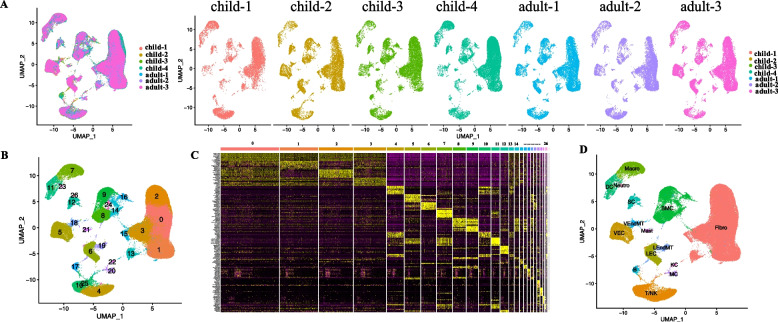
Cell clustering and cell-type identification of human prepuce based on scRNA-seq data. **A** UMAP plot showing 90,770 qualified prepuce cells from 4 children and 3 adults based on the top 2000 variable genes in expression. **B** Clustering results of the prepuce cells of children and adults. **C** Expression heatmap for the markers of 27 different cell clusters. **D** UMAP plot displaying the 15 distinct cell subtypes of prepuce cells

To explore the cell composition and expression heterogeneity of human prepuce at single-cell resolution, we first applied scRNA-seq to prepuce samples from 5 children and 4 adults. After removing the low-quality samples and cells (two unqualified samples of one child and one adult were excluded), a total of 90,770 qualified single cells were kept for 4 children and 3 adults (Fig. 1A). Based on the top 2000 variable genes, those prepuce cells from children and adults were mixed together in the UMAP plot (Fig. 1A). All these 7 samples showed similar cell distributions, suggesting that no batch effect exists among different samples. Using a graph-based clustering approach, the 90,770 cells of these 7 samples could be grouped into 27 different clusters with Seurat [21] (Fig. 1B). A series of marker genes with enriched expression were identified for different clusters (Fig. 1C, Supplementary Table S1). According to the known marker genes of corresponding cell types in CellMarker database [22], those 27 clusters could be further classified into 15 cell subtypes, including dendritic cells (DC), smooth muscle cells (SMC), fibroblast (Fibro), vascular endothelial cell (VEC), vascular endothelial-to-mesenchymal transition cell (VEndMT), lymphatic endothelial cell (LEC), lymphatic endothelial-to-mesenchymal transition cell (LEndMT), melanocyte (MC), keratinocyte/epithelium (KC/Epi), schwann cell (SC), Mast, Macrophages (Macro), neutrophils (Neutro), T/natural killer (NK) and B cells (Fig. 1D). Interestingly, fibroblast cells (55.52%) occupied the largest portion of cells, followed by SMC cells (10.49%), T/NK cells (9.50%), VEC (5.15%), Macro (4.93%), and LEC (4.83%).

### Characterization of marker expression and cell composition in the human prepuce

**Fig. 2:**
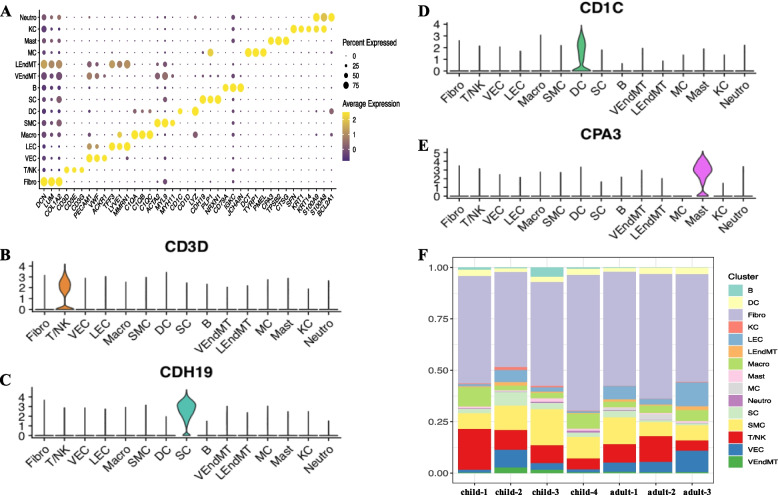
Maker expression profile and cell subtype composition of prepuce cells. **A** Expression distribution of selected markers for each type of prepuce cells. **B-E** Specific makers for T/NK, SC, DC, and Mast cell types. **F** Cell type composition of the prepuce in each individual of children and adults

For each of those 15 cell types, we identified a set of marker genes with enriched expression compared to other cell types (Fig. 2A, adjusted *p*-value < 0.05). For instance, DCN, LUM, and COL1A2 were highly expressed in fibroblast cells, while CD3D, CD3E, and CD3G showed enriched expression in T/NK cells (Fig. 2A). PECAM1, VWF, and ACKR1 were detected as the marker genes for VEC, while ACTA2, MYL9, and MYH11 exhibited high expression in SMC. CPA3, TPSB2, and CTSG were the marker genes of MAST, while SFN, KRT1, and KRT14 were with enriched expression in KC. Specifically, as shown in Fig. 2B-E, some marker genes like CD3D, CDH19, CD1C, and CPA3 were almost only expressed in T/NK, SC, DC, and Mast cells, respectively.

We further examined the cellular compositions of different prepuce cell subtypes in children and adults. For each individual of children and adults, fibroblast cells accounted for the largest portion of cells (children: 46.46%-66.18%; adults: 52.6%-60.78%). However, the ranking of other types of cells varied greatly among different individuals (Fig. 2F). For example, smooth muscle cells were the second most abundant cells for child-2 (11.85%), child-3 (17.51%), child-4 (10.37%), and adult-1 (13.11%), while T/NK cells occupied the second largest fraction for child-1 (19.98%) and adult-2 (12.68%). Interestingly, we also found that the proportions of all the 15 cell types were highly variable among the 4 children in general, whereas several cell types showed relatively constant small fractions (< 1%) among the 3 adults (e.g., B cells, KC, Mast, neutrophils, and VEndMT). These results suggest that human prepuce of both children and adults have prominent differences in composition ratios of cell types.

### Gene regulatory network inference for prepuce cells

**Fig. 3:**
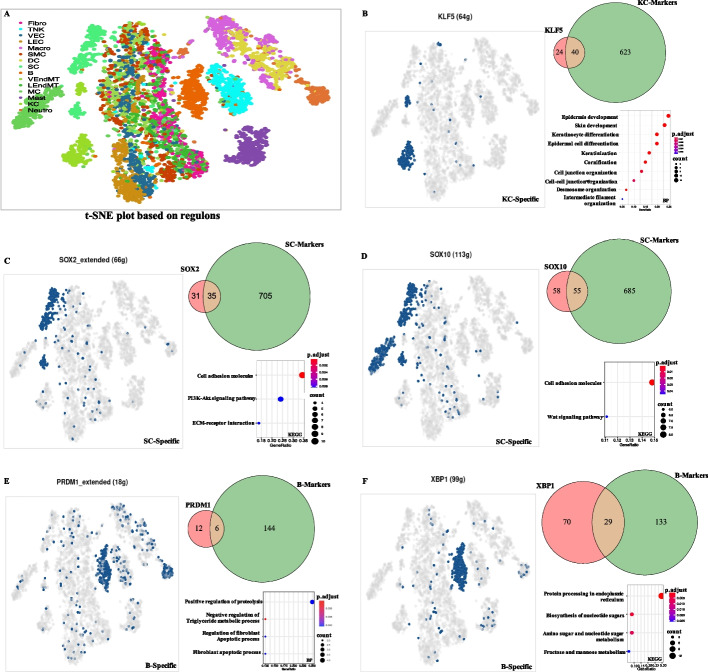
Gene regulatory network profile of human prepuce cells. **A** t-SNE plot showing the distribution of 15 cell types of prepuce based on 59 significant regulons. The cells in the plot were colored by corresponding cell types. **B-F** Profiles of cell-type specific regulons for KC, SC, and B cells, respectively. Venn plots displaying the intersection between downstream target genes of corresponding TF and the marker genes of related cell types. Bubble diagrams showing the enriched biological processes or KEGG pathways for the downstream target genes of the corresponding TF

To investigate the transcriptional regulatory profile of human prepuce, we inferred the gene regulatory networks (GRNs) in prepuce cells by employing SCENIC [23]. According to the expression associations between transcription factors (TFs) and downstream target genes, a total of 59 significant regulons were detected based on all 90,770 prepuce cells (each regulon contains one TF and its downstream target genes). These regulons involved 59 TFs and 2985 downstream target genes in total. As shown in Fig. 3A, different cell types can be distinguished in the t-SNE plot based on those 59 regulons, suggesting that the gene regulatory profiles of these cell types could be significantly different.

Intriguingly, we detected a set of cell-type-specific regulons that mainly activated in certain cell types. For example, the regulon formed by TF KLF5 was primarily detected in KC, which contained 64 downstream target genes (Fig. 3B). We observed that 40 out of those 64 (62.5%) downstream target genes of KLF5 were the marker genes of KC (e.g., EHF, KRT14, CALML5, SERPINB5, PKP1, and LGALS7), indicating the coregulation of these genes and the significant constribution of KLF5 to the expression profile of KC. Those 64 target genes of KLF5 were significantly enriched in the biological processes of epidermis development, skin development, keratinocyte differentiation, and epidermal cell differentiation (Fig. 3B), further demonstrating its important regulatory role in the development of human prepuce. The regulons formed by TF SOX2 (66 downstream target genes) and SOX10 (113 downstream target genes) were enriched in the cell subpopulations of SC (Figs. 3C and D). Of note, 35 out of those 66 (53%) SOX2 target genes (e.g., L1CAM, COL9A3, CADM4, AATK, MPZ, and ATP1A2) and 55 out of those 113 (48.7%) SOX10 target genes (e.g., GPR155, L1CAM, CNPY2, GFRA3, COL9A3, and CADM4) were the marker genes of SC. The enriched KEGG pathways for those 66 SOX2 downstream target genes were cell adhesion molecules, PI3K − Akt signaling pathway, and ECM − receptor interaction, while those 113 SOX10 downstream target genes were mainly enriched in cell adhesion molecules and Wnt signaling pathway (Fig. 3C and D). The regulons of TF PRDM1 (18 downstream target genes) and XBP1 (99 downstream target genes) were mainly detected in B cells (Fig. 3E and F). Compared with the B cell marker genes, 6 out of those 18 (33.3%) PRDM1 target genes (e.g., RAB30, TMEM156, PDK1, MIR155HG, FBXW7, and PRDM1) and 29 out of those 99 (29.3%) XBP1 downstream target genes (e.g., C16orf74, IRF4, TPD52, RAB30, CD79B, and DERL1) overlapped with those markers. Gene functional enrichment analysis showed that those 18 PRDM1 target genes were mainly involved in the biological processes of positive regulation of proteolysis, negative regulation of triglyceride metabolic process, regulation of fibroblast apoptotic process, and fibroblast apoptotic process, while those 99 XBP1 target genes were mainly enriched the KEGG pathways of protein processing in the endoplasmic reticulum, biosynthesis of nucleotide sugars, amino sugar and nucleotide sugar metabolism, and fructose and mannose metabolism (Fig. 3E and F). Therefore, those cell-type-specific regulons could play crucial roles in regulating the gene expression of corresponding cell subpopulations. The results would benefit the understanding of cellular expression heterogeneity and the function of different cell subtypes of the human prepuce.

### Dynamics of gene regulatory networks in human prepuce cells

**Fig. 4:**
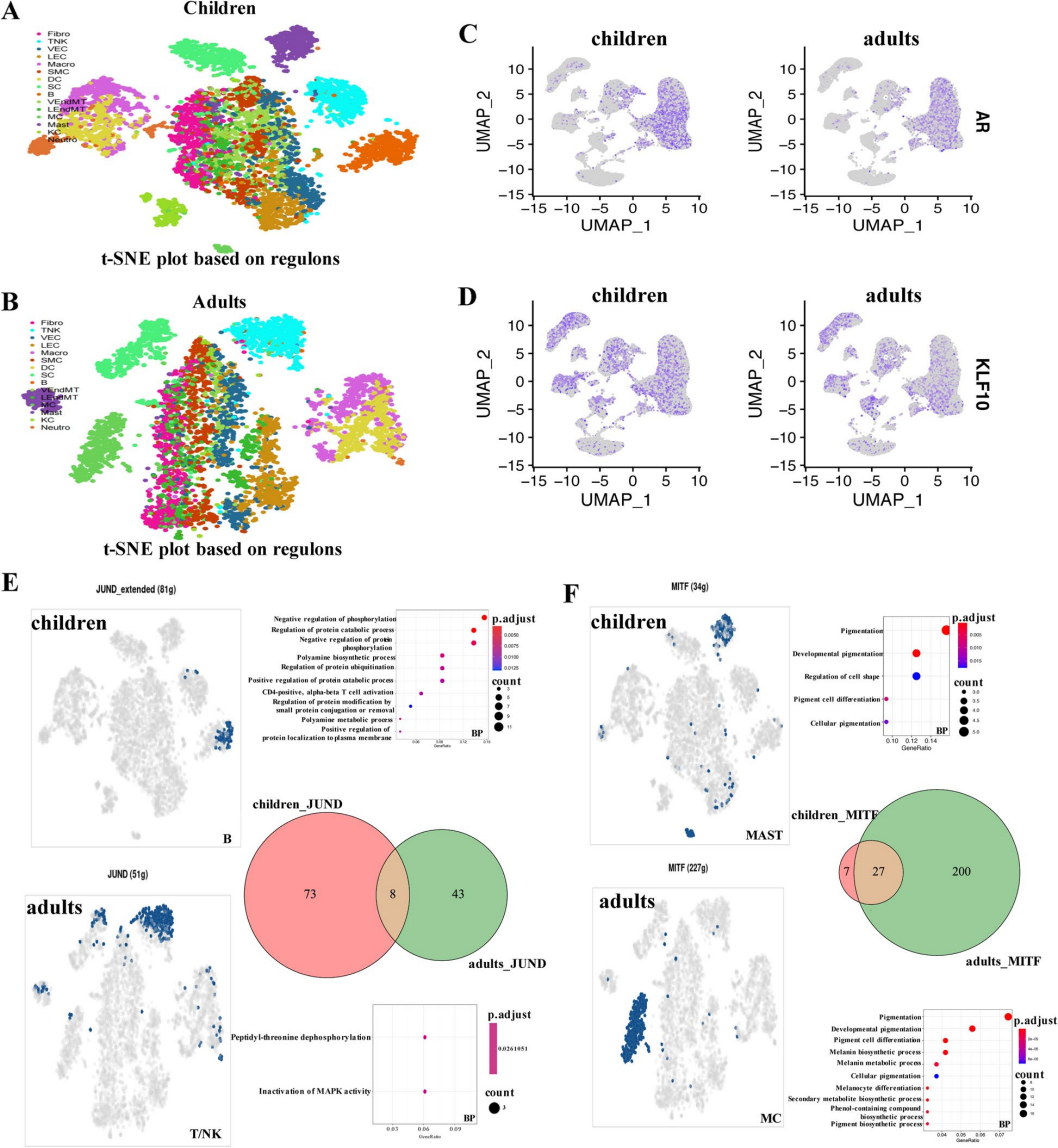
Gene regulatory differences between the prepuce cells of children and adults. **A** t-SNE plot of prepuce cells for children based on detected regulons. **B** t-SNE plot of prepuce cells for adults based on inferred regulons. **C** Expression profile of children-specific regulon AR. **D** Expression distribution of children-specific regulon KLF10. **E–F** Regulons with the same TF but having different sets of downstream target genes in children and adults. t-SNE plot showing the activation state of a regulon in cells (blue: activated; gray: un-activated). Venn plots displaying the intersection between the downstream target genes of the same TF-regulon in children and adults. The significantly enriched biological processes were shown in the bubble diagram

To gain insights into the transcriptional regulatory differences between children and adults, we further separately constructed the GRNs for these two groups. As shown in Figs. 4A and B, each cell type could be distinguished from other cell types based on the detected regulons for children and adults. Interestingly, we found that a few TFs formed potential age-specific regulons, which were also differentially expressed between children and adults. For example, TF androgen receptor (AR) formed a specific regulon (involved 19 downstream target genes, such as BNC2, BOC, and CRNDE) in children, which showed a more enriched expression in children compared to adults (Fig. 4C). AR is a steroid-hormone-activated TF and has the potential to affect cellular differentiation and proliferation in corresponding tissues [24]. TF KLF10 also showed higher expression in children than adults and formed a children-specific regulon (involved 19 downstream target genes, such as CNOT8, NFYA, and PHF21A) (Fig. 4D). Previous studies have shown that KLF10 might play a critical role in regulating the circadian clock [25, 26]. Accordingly, we identified potential children-specific regulons that were not detected in adults, these regulons could be important in regulating the expression of related gene sets in children's prepuce cells.

We also observed that several TFs formed corresponding regulons in both children and adults, but they activated in different cell types and the downstream target genes varied greatly. For instance, TF JUND formed a specific regulon in B cells of children (containing 81 downstream target genes), whereas it generated a different regulon (including 51 downstream target genes) specific to the T/NK cells of adults (Fig. 4E). Most of the downstream target genes regulated by JUND in children were different from those regulated in adults (Fig. 4E), only sharing 8 genes (CCND2, TNFRSF12A, ANXA6, JUND, BTG1, ODC1, DUSP8, and PRR7). Gene functional enrichment analysis showed that those downstream target genes regulated by JUND in children were mainly involved in the biological processes of negative regulation of phosphorylation, regulation of protein catabolic process, negative regulation of protein phosphorylation, and regulation of protein ubiquitination, while those JUND targeting genes in adults were enriched in peptidyl-threonine dephosphorylation and inactivation of MAPK activity. The regulon of TF MITF was specific to MAST cells of children (having 34 downstream target genes), whereas it formed a specific regulon (including 227 downstream target genes) in the adult MC (Fig. 4F). We found that the downstream target genes regulated by MITF had 27 common genes (such as CLCN7, SLCO4A1, GNPTAB, GNAL, and MSC) between children and adults (Fig. 4F). Those 34 MITF downstream target genes in children were mainly enriched in the biological processes of pigmentation, developmental pigmentation, regulation of cell shape, pigment cell differentiation, and cellular pigmentation, while those 227 downstream target genes regulated by MITF in adults were involved in similar biological processes as well (adjusted *p*-value < 0.05). Consequently, we revealed the dynamics of gene regulatory networks formed by the same TFs in prepuce cells between children and adults, which could contribute to the expression dynamics of human prepuce development and maintenance.

### Cell–cell communication network construction for human prepuce cells

**Fig. 5:**
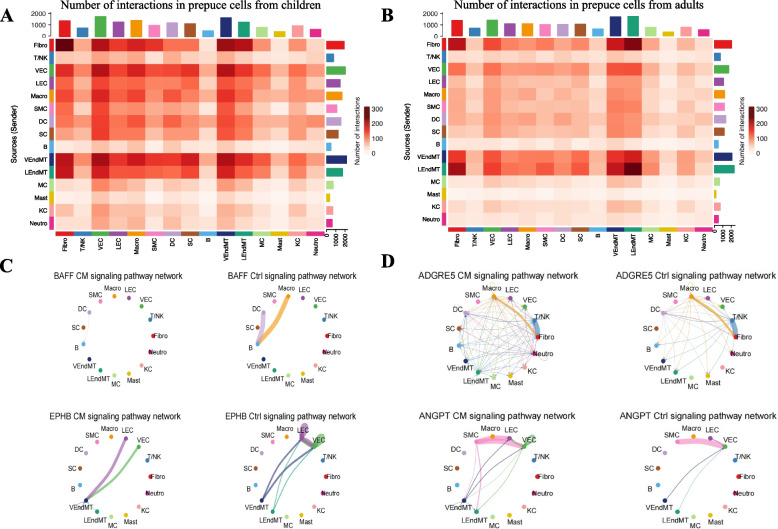
Cell–cell communication networks among different prepuce cell types in children and adults. **A** Cell–cell interaction heatmap between any two cell types of children prepuce. **B** Cellular communication heatmap between cell types for the prepuce of adults. **C** Signaling pathways that have stronger interaction intensity in children compared to adults. **D** Signaling pathways that show stronger interaction intensity in adults compared to children

To investigate the cellular communications among different cell types in children and adults, we constructed the cell–cell interaction networks based on the expression profiles of ligand-receptor pairs using CellChat [27]. By comparing the cell–cell interaction networks between children and adults, we found that fibroblast, VEndMT, and LEndMT cells exhibited relatively strong cellular communications with other cell types, whereas the cell types of T/NK, B, MC, Mast, KC, and Neutro showed relatively weak cell–cell interactions with other types of cells for both children and adults (Fig. 5A and B). However, cell types of VEC, LEC, Macro, SMC, DC, and SC generally had stronger cellular interactions with other types of cells in children compared to adults. For the outgoing cellular interaction strength, the largest was fibroblast in children followed by LEndMT, VEndMT, and LEC, whereas the strongest was LEndMT in adults followed by fibroblast, VEndMT, and DC (Supplementary Figure S1A). For the intensity of incoming cell–cell communications, the highest was VEC in children followed by VEndMT, Macro, and DC, while the largest was VEndMT in adults followed by LEndMT, VEC, and DC (Supplementary Figure S1B). For children, the lowest outgoing and incoming cellular interaction strength were KC and B cells respectively. In comparison, the weakest outgoing and incoming cell–cell interaction intensity were both Mast cells in adults.

We also found that the cellular interaction intensities for some enriched signaling pathways formed by corresponding ligand-receptor pairs were significantly different between children and adults (Fig. 5C and D). For example, stronger cell–cell interactions were observed between B cells and DC or Macro cells in children compared to adults for BAFF signaling pathway (Fig. 5C). Our result was in line with previous reports that the cytokine of BAFF was critical for supporting the survival of mature naïve B cells, which was also required for the survival of autoimmune B cells and memory B cells [28–30]. More cellular communications were also detected among VEndMT, LEC, and VEC in children than that in adults for the EPHB signaling pathway (Fig. 5C). Previous studies have shown that the EPHB signaling pathway plays an important role in cell adhesion and migration, indicating that it could promote the development of children's prepuce [31–34].

In contrast, some signaling pathways showed opposite trends between children and adults (Fig. 5D). For instance, the cell–cell interactions enriched in the ADGRE5 signaling pathway were stronger in adults compared to children. ADGRE5 encodes the proteins of the EGF-TM7 subfamily of adhesion G protein-coupled receptors, which may be functionally important for cell adhesion as well as the recruitment, activation, and migration of leukocytes [35–37]. A similar phenomenon was observed for the ANGPT signaling pathway, where higher cell–cell interaction intensity was detected among VEndMT, LEndMT, SMC, Macro, LEC, and VEC in adults compared to children. These signaling pathways could be important for prepuce maintenance in adults, reflecting the functional differences of cellular communications between children and adults.

### Cell–cell communication variations among different cell types of the human prepuce

**Fig. 6:**
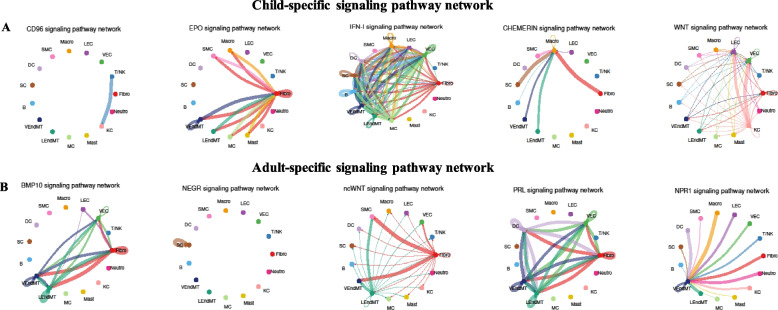
Enriched cell-type specific signaling pathways of children and adults. **A** Signaling pathways mediated by ligand-receptor pairs significantly enriched in children. **B** Signaling pathways significantly enriched in adults mediated by ligand-receptor pairs

We also detected the signaling pathways that were mainly enriched in children and adults, respectively. For example, the signaling pathways of CD96, EPO, IFN-1, CHEMERIN, and WNT were only identified as significant enrichment in children (Fig. 6A), suggesting that they could be mainly activated in children rather than adults. Cell–cell interactions were primarily detected between KC and T/NK cells for the CD96 signaling pathway. It has been suggested that CD96-mediated signaling had the potential to modulate the differentiation of effector T cells, which could have a co-stimulatory role in the activation and effector function of CD8 + T cells [38]. EPO signaling pathway was mainly enriched by the cell–cell interactions between fibroblast and other cell subtypes. Previous studies have revealed that EPO could enhance the differentiation of myofibroblasts, and also had the potential to accelerate skin wound closure [39–41]. CHEMERIN signaling pathway was mainly enriched by the interactions between Macro and other cell types, while IFN-I and WNT signaling pathways were mediated by the cell–cell communications among various cell types in the prepuce of children. Thus, these signaling pathways could be functionally important in the prepuce development of children.

The signaling pathways mainly detected in adults included BMP10, NEGR, ncWNT, PRL, and NPR1 (Fig. 6B). BMP10 signaling pathway was enriched by the cellular communications among the cell types of VEndMT, LEndMT, fibroblast, LEC, VEC, and T/NK, while the NEGR signaling pathway was mainly activated by the interactions with SC. The activation of the NPR1 signaling pathway in adults’ prepuce was primarily mediated by the cell–cell interactions between VEndMT and other cell types. By contrast, ncWNT and PRL signaling pathways were enriched by the cellular interactions among different cell types in adults. The cell–cell communications mediated by corresponding ligand-receptor pairs for these pathways could play an important role in the prepuce maintenance of adults. Our results indicate that the cell–cell interaction networks among different cell types varied greatly between children and adults.

## Discussion

To the best of our knowledge, we are the first to systematically explore the cellular heterogeneity of human prepuce between children and adults from different aspects (including expression, gene regulation, and cell–cell communication) at the single-cell level. A total of 15 different cell types were identified in human prepuce cells and each cell type had a set of marker genes with significantly enriched expression. Fibroblast cells accounted for the largest fraction in both children and adults. We observed that the proportion of those identified cell types in different individuals varied greatly in general. A number of differentially expressed genes (DEGs) between children and adults for each cell type were detected. The quantity of DEGs for those 15 cell types ranged from 3 to 981 (Supplementary Figure S2), indicating the large expression variation between the prepuce cells of children and adults.

We also revealed the dynamics of gene regulation among different cell types as well as between the prepuce of children and adults. In total, 59 significant regulons were detected based on the 90,770 prepuce cells, which were involved in 59 TFs and 2985 downstream target genes. Interestingly, we detected a number of cell-type-specific regulons with enriched activation for certain cell types, such as the KC-specific regulon formed by TF KLF5, the regulons of TFs SOX2 and SOX10 specific to SC, as well as the regulons of PRDM1 and XBP1 enriched in B cells (Fig. 3). Surprisingly, a large fraction of those downstream target genes regulated by these TFs were the marker genes detected for corresponding cell types, suggesting that cell-type-specific TFs played critical gene regulatory roles and could significantly affect the expression profile of related cell types. We further identified two regulons formed by TFs AR and KLF10 that were mainly activated in children prepuce compared to adults. Moreover, we also uncovered several regulons shared between children and adults but activated in different cell types and regulated distinct sets of downstream target genes. For instance, TF JUND formed a specific regulon in B cells of children but its regulon was enriched in T/NK cells of adults, while MITF regulon was mainly activated in MAST cells of children but it formed a specific regulon of MC in adults (Fig. 4E). Consequently, the prepuce cells showed high gene regulatory dynamics between children and adults. Our findings could facilitate a better understanding of expression heterogeneity of prepuce cells during development.

We constructed the cell–cell communication networks among different cell types of the human prepuce and discovered large-scale variations between children and adults. Strong cell–cell interactions were observed among Fibroblast, VEndMT, and LEndMT cells, while relatively weak cellular communications were detected among T/NK, B, MC, Mast, KC, and Neutro. Furthermore, we also observed that some enriched signaling pathways exhibited highly different cellular interaction intensities between children and adults, such as BAFF, ADGRE5, EPHB, and ANGPT signaling pathways. Additionally, we identified a number of signaling pathways specific to children (e.g., CD96, EPO, IFN-1, CHEMERIN, and WNT signaling pathways) and adults (e.g., BMP10, NEGR, ncWNT, PRL, and NPR1 signaling pathways). Our results not only showed the cellular communication variations among different cell types between children and adults, but also revealed the signaling pathways that could contribute to the prepuce development in children and the prepuce maintenance in adults.

Given the results obtained from our study, several avenues for future investigations could be conducted. One promising direction is the incorporation of spatial transcriptomics techniques to complement our understanding of the prepuce's cellular organization and interactions within its microenvironment. Spatial transcriptomics can provide insights into the spatial distribution of cell types and their gene expression patterns [42–44], providing additional information for cell–cell communication networks and tissue organization within the prepuce. On the other hand, the ages of the four children (4, 4, 5, and 5) are very similar in this study, the age differences of adults (19, 32, and 37) may introduce certain biological variations. Further investigation of the longitudinal changes in cell heterogeneity across more groups with similar ages could facilitate better understanding of the development and maintenance of human prepuces. Additionally, exploring the prepuce's cellular dynamics in disease states may provide valuable insights into its tissue homeostasis and susceptibility to pathological conditions.

In conclusion, we dissected the prepuce cells in terms of cellular compositions, gene expression changes, gene regulatory dynamics, and cell–cell communication variations, which shed light on the cellular heterogeneity and underlying molecular mechanisms of human prepuce development and maintenance.

## Materials and methods

### Quality control of scRNA-seq data and cell type identification

The scRNA-seq approach of 10X Genomics was applied to the prepuce samples from 5 children and 4 adults. Two samples from one child and one adult were excluded due to the low quality of scRNA-seq data. The remaining qualified scRNA-seq data of 4 children (ages of 4, 4, 5, and 5) and 3 adults (ages of 19, 32, and 37) were used in the downstream analysis. We further employed Seurat (version 4.1.1) [21] to conduct cell quality control, dimensionality reduction, and clustering. Those cells with > 5,000 or < 200 expressed genes, > 10% mitochondrial genes, or > 0.01% red blood cell genes were removed. Then we used the function of FindVariableFeatures in Seurat to select the top 2000 variable genes in expression for dimensionality reduction. The function of FindClusters in Seurat was applied to group cells into different clusters with Uniform Manifold Approximation and Projection (UMAP). We determined the cell types of prepuce with corresponding cluster markers using the CellMarker database [22]. Specifically, the top 10 marker genes detected in each cell cluster were compared with the known makers of related cell types in CellMarker database. According to the overlap of marker genes, the cell clusters were grouped into corresponding cell types.

### Gene functional enrichment analysis

We employed the R package of clusterProfiler [45] (version 3.18) to carry out Gene Ontology (GO) and KEGG pathway enrichment analysis. Adjusted *p*-value < 0.05 was used to define the significantly enriched biological processes and KEGG pathways. Gene set enrichment analysis (GSEA) was based on the Molecular Signatures Database (http://www.gsea-msigdb.org/gsea/index.jsp).

### Inference of single-cell gene regulatory networks

Single-cell gene regulatory networks of cells formed by transcription factors (TFs) and their downstream target genes were inferred using SCENIC (version 1.1.2) [23]. Specifically, the normalized gene expression matrix of cells was first used as the input for the R package of GENIE3 (version 1.16.0) to construct co-expression networks of genes. Next, RcisTarget (version 1.14.0) was employed to deduce the regulatory networks between TFs and downstream target genes. After the construction of gene regulatory networks, AUCell (version 1.16.0) was utilized to analyze the activity of predicted regulons in each cell. Each regulon was formed by the TF and its downstream target genes. Adjusted *p*-value < 0.05 was applied to define the significance of regulons.

### Cell–cell communication network construction and signaling pathway analysis

We used CellChat (version 1.4.0) [27] to construct the cell–cell interaction network among different cell types of human prepuce. Cellular communications between two different cell types or within the same cell types were predicted based on the expression profile of known ligand-receptor pairs. CellChat computed an enrichment score for each potential ligand-receptor interaction by comparing the joint expression of the ligand in the sending cell type and the receptor in the receiving cell type against their individual background expression distributions. Statistically significant interactions between cell types were identified based on empirical p-values using a permutation-based test. By summarizing the probabilities of ligand-receptor pairs, the communication probability of a signaling pathway is computed by CellChat. Using manifold learning and quantitative comparisons, CellChat classified signaling pathways and identified conserved and context-specific pathways for children and adults.

### Supplementary Information

**Additional file 1:** **Supplementary** **Table S1.** Top 10 markers in each cell cluster used for determining corresponding cell types.

**Additional file 2.**

**Additional file 3.**

## Data Availability

All raw and processed sequencing data generated in this study have been submitted to the National Omics Data Encyclopedia (NODE, https://www.biosino.org/node/) database under accession number OEX020875.
