# Supplementary material for: Single-cell analysis of human prepuce reveals dynamic changes in gene regulation and cellular communications
Source: BMC Genomics. 2023 Sep 1;24:514. doi: 10.1186/s12864-023-09615-8 (PMC10474653; doi:10.1186/s12864-023-09615-8)
Supplement: Supplementary file 1 — Additional file 1: Supplementary Table S1. Top 10 markers in each cell cluster used for determining corresponding cell types. [file 12864_2023_9615_MOESM1_ESM.docx]

**Table 1.** Top 10 markers in each cell cluster used for determining corresponding cell types.

| **Cluster** | **Maker gene** | | | | | | | | | | **Cell type** |
| --- | --- | --- | --- | --- | --- | --- | --- | --- | --- | --- | --- |
| 0 | PTGDS | SFRP2 | COL1A2 | CXCL14 | COL1A1 | LUM | DCN | FBLN1 | MGP | PCOLCE2 | Fibro |
| 1 | APCDD1 | SERPINE2 | DIO2 | IGFBP2 | APOE | TWIST2 | CRABP1 | PTGDS | IGFBP4 | COL6A1 | Fibro |
| 2 | MFAP5 | IGFBP6 | FBN1 | IGFBP5 | FN1 | CD55 | SEMA3C | C17orf58 | PI16 | NOVA1 | Fibro |
| 3 | MGP | APOC1 | APOE | CFH | APOD | CXCL12 | GPC3 | IGF1 | IGFBP7 | GSN | Fibro |
| 4 | CCL5 | NKG7 | DUSP2 | CCL4 | CD69 | IL32 | XCL1 | CXCR4 | HCST | XCL2 | T/NK |
| 5 | ACKR1 | IFI27 | AQP1 | VWF | PECAM1 | SELE | A2M | EGFL7 | CLEC14A | PLVAP | VEC |
| 6 | CCL21 | MMRN1 | TFF3 | CLDN5 | PPFIBP1 | GNG11 | LYVE1 | CAVIN2 | TM4SF1 | PKHD1L1 | LEC |
| 7 | RNASE1 | C1QA | C1QB | C1QC | CCL18 | CXCL8 | F13A1 | LGMN | FOLR2 | CCL3 | Macro |
| 8 | RGS5 | CCL2 | TAGLN | STEAP4 | FABP4 | ID4 | RGS16 | NDUFA4L2 | C2orf40 | ACTA2 | SMC |
| 9 | RERGL | ACTA2 | TAGLN | TPM2 | MYH11 | MYL9 | C11orf96 | MCAM | SORBS2 | BCAM | SMC |
| 10 | IL32 | IL7R | TRBC2 | PTPRC | CD52 | CD2 | CD3D | LTB | SAMSN1 | CD3G | T/NK |
| 11 | LYZ | HLA-DPB1 | HLA-DRA | HLA-DPA1 | HLA-DQB1 | HLA-DRB1 | HLA-DQA1 | IL1B | HLA-DRB5 | CD74 | DC |
| 12 | NRXN1 | S100B | CDH19 | CRYAB | GPM6B | SCN7A | MPZ | PLP1 | VWA1 | CD9 | SC |
| 13 | IL32 | CD52 | CXCR4 | CCL5 | IL7R | DUSP2 | CD3D | RGCC | CD69 | RUNX3 | Fibro |
| 14 | RGS5 | TAGLN | ACTA2 | C11orf96 | CALD1 | TPM2 | MYL9 | MT1A | NR2F2 | NOTCH3 | SMC |
| 15 | APOD | FGFBP2 | CLDN1 | FOXS1 | C2orf40 | NRP2 | SCN7A | KLF5 | KLK1 | NOV | Fibro |
| 16 | DES | MYH11 | ACTG2 | TAGLN | MYLK | TPM2 | MYL9 | ACTA2 | CNN1 | PCP4 | SMC |
| 17 | IGKC | IGLC2 | JCHAIN | IGHA1 | IGHM | IGHG1 | IGHG3 | IGHG4 | IGLC3 | IGHA2 | B |
| 18 | RGS5 | IFI27 | AQP1 | A2M | NDUFA4L2 | ADGRF5 | RBP7 | PLVAP | CLEC14A | PALMD | VEndMT |
| 19 | CCL21 | TFF3 | MMRN1 | CLDN5 | PPFIBP1 | CAVIN2 | LYVE1 | TM4SF1 | PROX1 | LMO2 | LEndMT |
| 20 | DCT | TYRP1 | PMEL | MLANA | MFSD12 | CYB561A3 | MITF | QPCT | TRPM1 | KIT | MC |
| 21 | TPSB2 | CTSG | TPSAB1 | HPGD | CPA3 | KIT | CMA1 | GCSAML | HPGDS | MS4A2 | Mast |
| 22 | S100A7 | S100A8 | KRT1 | S100A9 | KRT14 | KRTDAP | CALML5 | KRT5 | KRT13 | DMKN | KC |
| 23 | S100A9 | S100A8 | CXCL8 | BCL2A1 | MNDA | IL1B | C5AR1 | FCGR3B | CSF3R | AQP9 | Neutro |
| 24 | CD3D | TAGLN | ACTA2 | CD3G | CD3E | CD52 | CD2 | TRBC2 | CD69 | TINAGL1 | SMC |
| 25 | TFF3 | MMRN1 | PKHD1L1 | TBX1 | CD3E | CLDN5 | TRBC2 | SCN3B | ECSCR | MPP7 | T/NK |
| 26 | NRXN1 | XKR4 | CDH19 | CHL1 | PCSK2 | COL28A1 | GFRA3 | S100B | MPZ | AL121820.2 | SC |
