# Supplementary figures and images for: Single-cell analysis of human prepuce reveals dynamic changes in gene regulation and cellular communications
Source: BMC Genomics. 2023 Sep 1;24:514. doi: 10.1186/s12864-023-09615-8 (PMC10474653; doi:10.1186/s12864-023-09615-8)

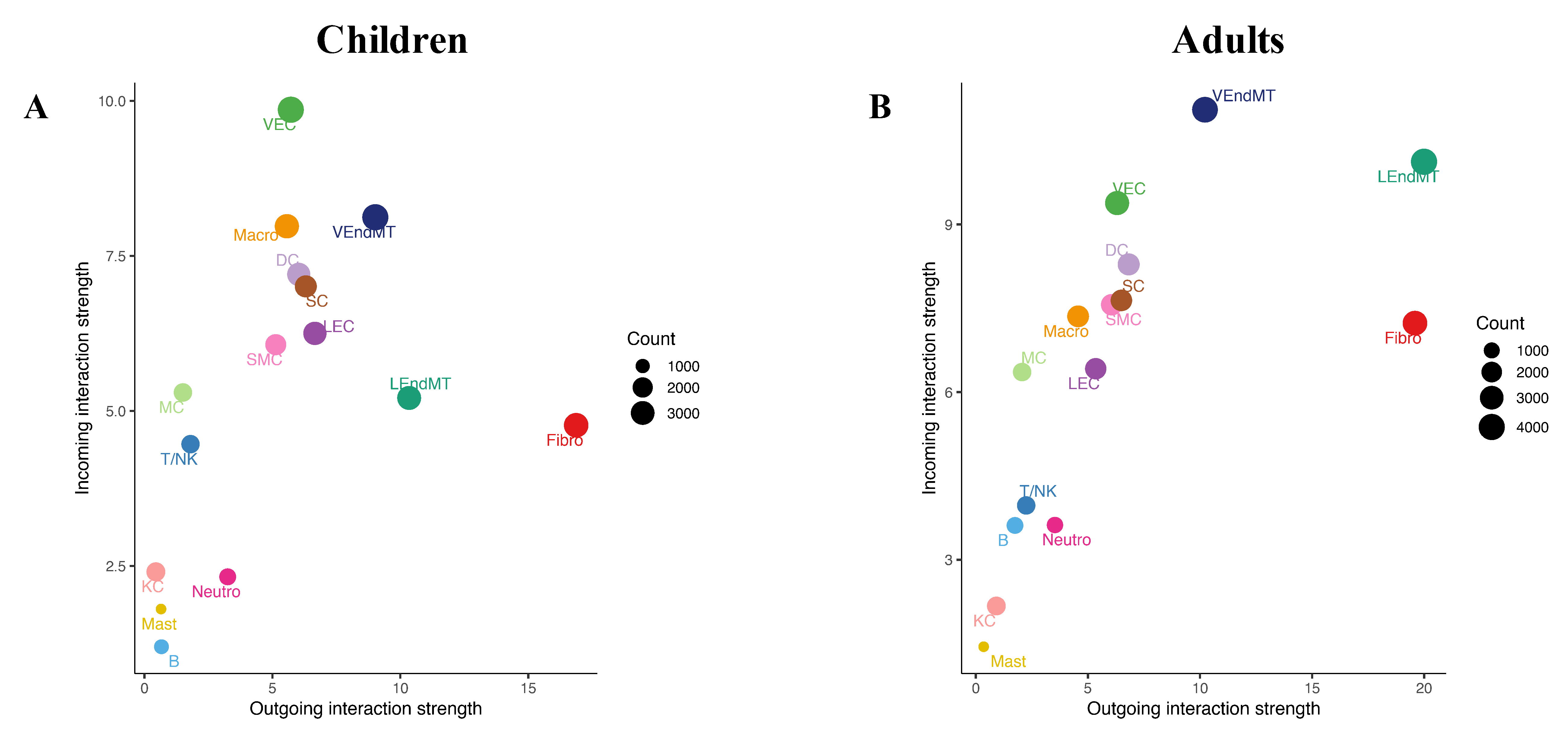

Supplement: Supplementary file 2 — Additional file 2. [file 12864_2023_9615_MOESM2_ESM.tiff]

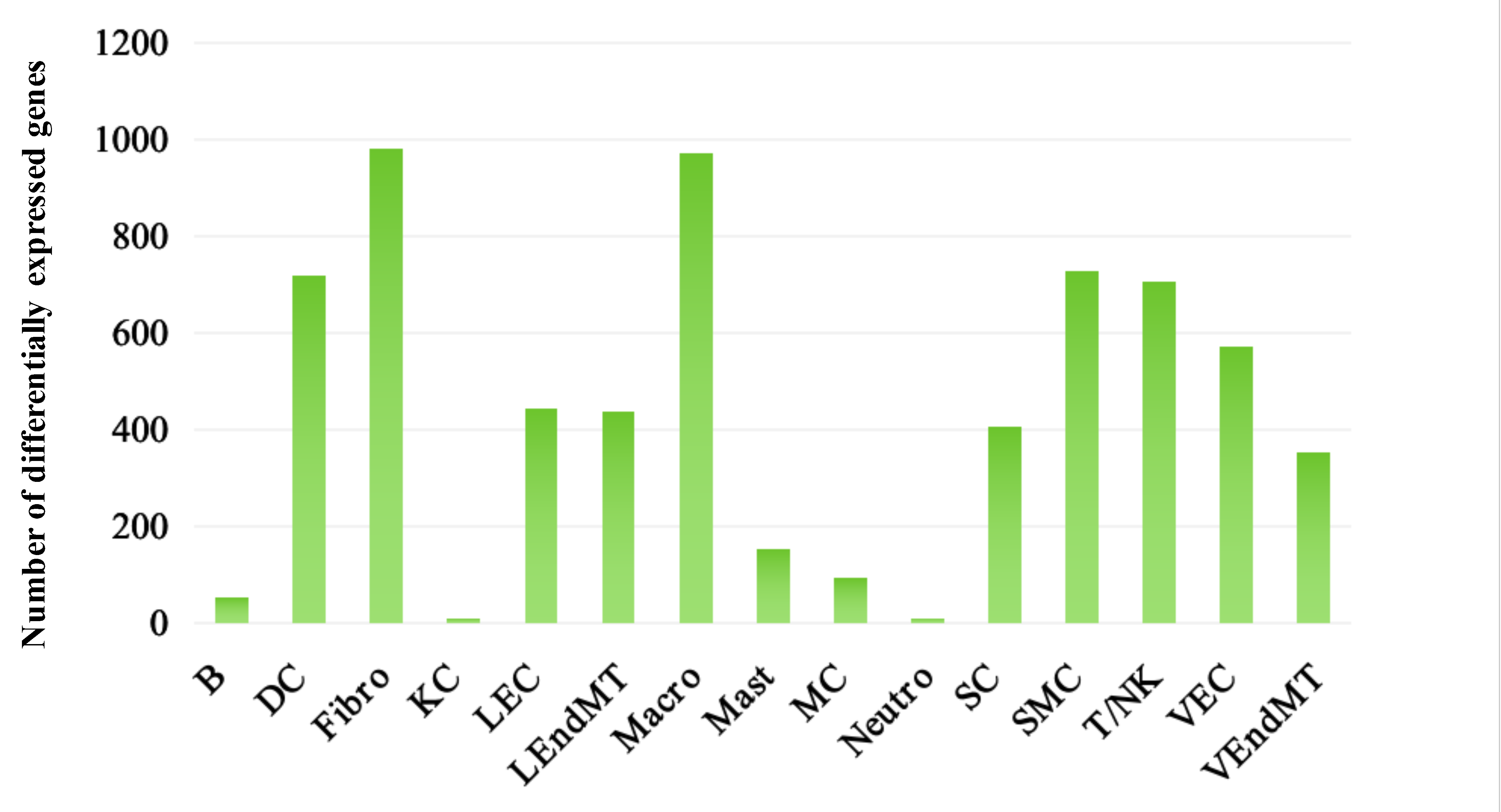

Supplement: Supplementary file 3 — Additional file 3. [file 12864_2023_9615_MOESM3_ESM.tiff]
